# Impact of Donor Substance Use on Outcomes Following Pancreas Transplantation

**DOI:** 10.1111/ctr.70722

**Published:** 2026-10-22

**Authors:** Sarah Huber, Richard S. Mangus, Quinton J. Mangus, Andrew J. Lutz, John A. Powelson, Jonathan A. Fridell

**Affiliations:** ^1^ Department of Surgery Indiana University School of Medicine Indianapolis Indiana USA; ^2^ Surgical Outcomes and Quality Improvement Center (SOQIC), Department of Surgery Indiana School of Medicine Indianapolis Indiana USA

**Keywords:** donor substance use, outcomes, pancreas transplant

## Abstract

**Background:**

Donor substance use is considered by many a risk factor for poor outcomes following pancreas transplantation, yet little is known regarding the impact of donor substance use on actual outcomes. This study aimed to determine the impact of substance use, particularly cocaine, on early and late outcomes following pancreas transplantation.

**Methods:**

This was a single center, retrospective analysis of all pancreas transplants from 2003‐2023. Clinical outcomes including 30‐day peak serum lipase, 7‐day, 90‐day, and 1‐year pancreas allograft survival were assessed by donor exposure to substance use. As cocaine was of particular interest, a Kaplan‐Meier analysis for positive vs negative cocaine tox screen was performed.

**Results:**

737 pancreas transplants were included. Donor substance use history included >20 pack years tobacco (40%) and marijuana (31%), with 11% of donors having history of cocaine use. Donor toxicology screens were positive for THC (26%), EtOH (22%), and benzodiazepines (22%). History of heavy alcohol use was associated with early allograft loss (7‐day allograft loss 8% compared to 3%, *p* < 0.01) and EtOH positive toxicology screen was associated with higher 90‐day allograft loss (8% vs 5%, p 0.02) and worse 1‐year allograft survival (89% vs 93%, *p* < 0.01). There was no difference in clinical outcomes with donor exposure to cocaine.

**Conclusions:**

Only a donor history of ETOH use was associated with worse outcomes following pancreas transplantation. Donor exposure to cocaine was not associated with inferior outcomes. Donor substance use history or positive toxicology in the setting of an otherwise acceptable pancreas allograft, should not preclude transplantation.

**Abbreviations**
DBD: Deceased after brain death donor
DCD: Deceased after cardiac death
EtOH: Alcohol
IVDU: Intravenous drug use
OPO: Organ Procurement Organization
OPTN: Organ Procurement and Transplantation Network
PAK: Pancreas after Kidney Transplant
PTA: Pancreas Transplant Alone
SPK: Simultaneous Pancreas Kidney Transplant
THC: Tetrahydrocannabinol

## 1 Introduction

Donor pancreas allografts available for transplant continue to fall short of waitlist demands. There are concerted efforts to increase the available donor pool, including reassessment of risk tolerance for donor characteristics previously viewed as high risk [1], such as donor substance use. Donor history of substance misuse, whether of alcohol, tobacco, or illicit drugs, may contribute to pancreas allograft non‐utilization, despite limited evidence regarding the impact of donor substance use on pancreas transplant outcomes. Anecdotally, it seems that donor history of cocaine use is considered particularly concerning.

The impact of various substances on outcomes of other solid organ transplants has been evaluated. Previous literature demonstrated that donor history of alcohol use does not negatively impact outcomes following liver transplant [2], however allografts from donors with history of cocaine use were associated with poorer short‐term outcomes [3]. Specifically for kidney allografts, positive donor toxicology screening does not impact graft failure or delayed graft function [4], though donor history of cigarette use has been associated with decreases in both graft survival and recipient survival [5]. Similarly, literature supports the use of hearts and lungs from donors with history of substance use [6, 7, 8, 9], though the impact of donor nicotine use on heart transplant outcomes is less clear [10].

Whether donor substance use influences outcomes following pancreas transplantation remains poorly understood. Given the persistent shortage of pancreas allografts and the certainty that transplant centers will receive at least some organ offers from donors with substance use, better understanding of the clinical significance of donor substance use is needed. This study was designed to evaluate the association between reported donor substance use history and positive toxicology findings with early and late outcomes following pancreas transplantation, with particular attention to cocaine exposure.

## 2 Methods

### 2.1 Study Design and Population

This was a single center, retrospective analysis of all adult deceased donor pancreas transplants performed at Indiana University from January 2003 to December 2023. Data were collected from the comprehensive transplant recipient registry maintained by our institution, individual electronic medical records, and the original donor medical history. The institutional review board of the Indiana University School of Medicine approved retrospective review of data from the transplant center. Inclusion criteria were pancreas allograft implantation as an independent organ, including simultaneous pancreas and kidney transplant (SPK), pancreas transplant after kidney transplant (PAK), pancreas transplant alone (PTA), and pancreas retransplant. Recipients of transplants in which the pancreas was transplanted en bloc were excluded, such as multivisceral transplants. All recipients listed for transplantation at Indiana University were in accordance with standard protocols and procedures, defined by our center and the Organ Procurement and Transplantation Network (OPTN). Although donor substance use may have influenced the decision of whether or not to accept a pancreas allograft, this history did not influence the selection of which recipient would receive the accepted allograft.

Standard pancreas allograft procurement followed aortic flush with preservation solution and topical cooling with saline slush [11, 12]. In all cases the pancreas was examined in situ during retrieval and again on the backbench. Allografts were only transplanted if the allograft appeared acceptable without excessive edema or firmness. The pancreas was routinely positioned with the head and duodenum oriented superiorly and the tail directed inferiorly toward the pelvis. The infrarenal inferior vena cava or right common iliac vein were utilized for systemic venous drainage. A stapled technique was used to establish enteric drainage [13]. For those transplants that included a kidney, pulsatile perfusion was routinely used for the renal allograft [14, 15] and the preferred placement was ipsilateral placement of both allografts [16].

The immunosuppression induction regimen consisted of five doses of rabbit antithymocyte globulin (rATG) (1 mg/kg/dose) with steroid premedication [17]. Maintenance immunosuppression consisted of tacrolimus (target trough level 6‐8 mCg/mL) and sirolimus (target trough level 3‐6 mCg/mL), and with the addition of mycophenolic acid 360 mg BID for PTA recipients [17]. No steroids were utilized for maintenance immunosuppression, and all steroids were discontinued following induction [18]. This institutional immunosuppression regimen has been in place since 2002. All recipients received routine perioperative antibiotics as per institutional protocols, as well as prophylaxis against cytomegalovirus and *Pneumocystis jirovecii*, with oral valganciclovir and trimethoprim/sulfamethoxazole respectively. Systemic anticoagulation was not routinely used, but patients did receive deep venous thrombosis prophylaxis with heparin or enoxaparin depending on early renal function.

### 2.2 Variables

Donor history of substance use was collected from the local Organ Procurement Organization (OPO) database or Donornet, including data from medical records, donor urine toxicology screens, and reported history of substance use obtained from next of kin in the Uniform Donor Risk Assessment Interview. Donor history of substance use included alcohol, tobacco, marijuana, intravenous drug use (IVDU), and cocaine. Alcohol use history was considered positive if more than 2 drinks/day for female donors and 3 drinks/day for male donors or history of binge drinking [19]. Tobacco use was considered positive if equivalent to or greater than 20 pack years. History of cocaine use was stratified by any history of use and history of use within the past 6 months. Donor toxicology screens routinely tested for ethanol, tetrahydrocannabinol (THC), amphetamine, barbiturate, cocaine, opiates, benzodiazepines, and methadone. Note that toxicology screening is not a test that is required by the OPTN, so would only have been performed at the discretion of the managing medical team and would therefore not be available for all donors—but would certainly have been performed if there was a suspicion of substance use or misuse playing a role in the donor's cause of death. While marijuana use does result in positive toxicology screen for THC, other substances such as hemp products or CBD oil may result in a positive for THC, thus we did not reclassify positive THC as positive for marijuana. The donor history questionnaire specifically uses the term marijuana. Other covariates of interest included transplant type (SPK vs PAK vs PTA or retransplant), recipient and donor gender, race, age, and BMI, donor type (donation after brain death (DBD) vs donation after cardiac death (DCD)), donor cause of death (stroke, trauma, anoxic brain injury), and total ischemia time (hours).

### 2.3 Outcomes

Primary transplant outcomes included 30‐day peak serum lipase, 30‐day peak serum amylase, and 7‐day, 90‐day, and 1‐year pancreas allograft survival. Secondary outcomes included hospital length of stay and readmissions within one‐year post‐transplant, stratified as any and total number of readmissions. Pancreas allograft failure was defined as allograft pancreatectomy, relisting for pancreas transplantation, dependence on subcutaneous insulin administration, or death with a functioning allograft.

### 2.4 Statistical Analysis

Statistical analysis was conducted using commercially available software (IBM SPSS). Donor and recipient characteristics were analyzed using chi square for categorical variables and ANOVA for continuous variables, with differences considered to be significant for *p*‐value <0.05. As cocaine was of particular interest, a Kaplan‐Meier analysis for positive versus negative toxicology screen for cocaine was performed.

## 3 Results

### 3.1 Study Population

There were 737 pancreas transplants performed, of which there were 451 SPK (61%), 113 PAK (15%), and 173 PTA (24%). Of these,51 were retransplants (7%). Donor and recipient demographics for donors with a history of substance use and for positive donor toxicology screen are presented in Tables 1 and 2, respectively. Reported donor substance use history was available from 727 donors (98.1%). The most common substances that donors had a history of using were tobacco (40%) and marijuana (31%). Of the donors with a history of cocaine use (11%), nearly half had a history of use within the past 6 months (45.5% of all donors with a history of cocaine use). Additionally, 9% of donors had a history of alcohol use. In this population, a history of IVDU was rare, with only 18 (3%) donors identified. Toxicology screens were available for 463 (63%) of donors and were most commonly positive for THC (26%), ethanol (22%) and benzodiazepines (22%), although the latter may have been administered in some patients during the initial treatment phase. Donors with a history of alcohol use were older (median age 33 vs 24, *p* < 0.001) compared to the overall cohort. Allografts from donors with a history of tobacco use were more frequently from Black donors (11% vs 14%, *p* < 0.01), older donors (median age 27 vs 24, *p* < 0.001), and more often with death caused by stroke (18% vs 15%, *p* = 0.04). Allografts from donors with a history of marijuana use were more often transplanted into male recipients (71% vs 63%, *p* < 0.01). Donors with a history of IVDU more frequently died of anoxic brain injury (59% vs 26%, *p* < 0.01). Donors with a history of cocaine use were more likely to be DCD donors and more likely to have died from anoxic brain injury when compared to the overall cohort, both with any history of cocaine use and with history of cocaine use in the last 6 months. Recipient characteristics by donor history of substance use were largely similar between the groups, with no significant differences in age or BMI (Table 1). SPK recipients were less likely to have a donor with a history of tobacco use (57% vs 61%, *p* = 0.01). Recipients of allografts from donors with history of alcohol use or any cocaine use were more likely to be male. Black recipients were more likely to have a donor with a history of recent cocaine use (22% vs 9%, *p* = 0.03).

**TABLE 1:** Characteristics of pancreas transplant recipients, stratified by donor substance use history and donor toxicology screen.

History of Donor Substance Use													
	*N* (%)	Ethanol	*p*	Tobacco	*p*	Marijuana	*p*	IVDU	*p*	Cocaine (any)	*p*	Cocaine (last 6 months)	*p*
**Total Cohort**	737 (100%)	63 (9%)		285 (40%)		216 (31%)		18 (3%)		75 (10%)		36 (5%)	
**Transplant type**			0.98		0.01		0.41		0.85		0.98		0.25
SPK	451 (61%)	38 (61%)		162 (57%)		133 (62%)		12 (67%)		46 (61%)		20 (56%)	
PAK	113 (15%)	10 (16%)		57 (20%)		38 (18%)		2 (11%)		12 (16%)		9 (25%)	
PTA	173 (24%)	15 (24%)		66 (23%)		45 (21%)		4 (22%)		17 (23%)		7 (19%)	
**Retransplant**	51 (7%)	6 (10%)	0.56	23 (8%)	0.25	15 (7%)	0.63	3 (17%)	<0.001	5 (7%)	0.89	4 (11%)	0.53
**Gender**			< 0.01		0.64		1		0.63		0.01		0.04
Male	414 (56%)	45 (71%)		162 (57%)		122 (56%)		11 (61%)		52 (69%)		26 (72%)	
Female	323 (44%)	18 (29%)		123 (43%)		94 (44%)		7 (39%)		23 (31%)		10 (28%)	
**Race**			0.44		0.78		0.70		0.52		0.32		0.03
White	657 (89%)	55 (87%)		256 (90%)		190 (88%)		15 (83%)		65 (87%)		28 (78%)	
Black	69 (9%)	8 (13%)		26 (9%)		24 (11%)		3 (17%)		10 (13%)		8 (22%)	
Other	11 (1%)	0 (0%)		3 (1%)		2 (1%)		0 (0%)		0 (0%)		0 (0%)	
**Age** (median, (SE))	43 (0.4)	42 (1.3)	1.00	44 (0.6)	0.41	42 (0.6)	0.33	41 (2.6)	0.87	44 (1.2)	0.47	43 (1.6)	0.60
**BMI** (median, (SE))	25.2 (0.2)	25.4 (0.6)	0.69	25.0 (0.3)	0.82	25.4 (0.3)	0.96	28.5 (1.1)	0.09	26.1 (0.4)	0.21	25.8 (0.5)	0.93

Positive Donor Toxicology Screen																			
	*N* (%)	Toxicology Screen Obtained	*p*	EtOH	*p*	THC	*p*	Amphetamine	*p*	Barbiturate	*p*	Cocaine	*p*	Opiate	*p*	Benzodiazepine	*p*	Methadone	*p*
**Total Cohort** (*n* (%))	737	463 (63%)		96 (22%)		126 (26%)		29 (6%)		10 (2%)		27 (6%)		53 (11%)		106 (22%)		5 (1%)	
**Transplant type** (*n* (%))		<0.001			0.19		0.10		0.69		0.42		0.74		0.83		0.64		0.33
SPK	451 (61%)	301 (65%)		58 (60%)		90 (71%)		17 (59%)		5 (50%)		16 (59%)		33 (62%)		71 (67%)		5 (100%)	
PAK	113 (15%)	56 (12%)		15 (16%)		16 (13%)		4 (14%)		1 (10%)		4 (15%)		7 (13%)		14 (13%)		0 (0%)	
PTA	173 (24%)	106 (23%)		23 (24%)		20 (16%)		8 (28%)		4 (40%)		7 (26%)		13 (25%)		21 (20%)		0 (0%)	
**Retransplant** (*n* (%))	51 (7%)	32 (7%)	0.80	7 (7%)	0.56	6 (5%)	0.60	2 (7%)	0.92	0 (0%)	0.71	2 (7%)	0.90	2 (4%)	0.68	12 (11%)	0.02	0 (0%)	0.86
**Gender** (*n* (%))			<0.01		0.53		0.88		0.71		0.17		0.65		0.02		0.96		0.94
Male	414 (56%)	278 (60%)		60 (63%)		73 (58%)		18 (62%)		8 (80%)		17 (63%)		39 (74%)		62 (59%)		3 (60%)	
Female	323 (44%)	185 (40%)		36 (38%)		53 (42%)		11 (38%)		2 (20%)		10 (37%)		14 (26%)		44 (42%)		2 (40%)	
**Race** (*n* (%))			0.20		0.81		0.02		0.62		0.49		0.03		0.56		0.6		0.69
White	656 (89%)	412 (89%)		85 (89%)		104 (83%)		25 (86%)		10 (100%)		20 (74%)		47 (89%)		92 (87%)		5 (100%)	
Black	69 (9%)	42 (9%)		10 (10%)		21 (17%)		4 (14%)		0 (0%)		5 (19%)		6 (11%)		13 (12%)		0 (0%)	
Other	11 (1%)	9 (2%)		1 (1%)		1 (1%)		0 (0%)		0 (0%)		2 (7%)		0 (0%)		1 (1%)		0 (0%)	
**Age (median, (SE))**	43 (0.4)	42 (0.5)	0.69	41 (1.0)	0.73	43 (0.9)	0.43	44 (1.9)	0.96	41 (3.7)	0.40	40 (2.0)	0.67	44 (1.4)	0.87	42 (1.0)	0.61	35 (6.6)	0.85
**BMI (median, (SE))**	25.2 (0.2)	25.4 (0.2)	0.12	25.0 (0.5)	0.52	25.5 (0.4)	0.92	25.6 (0.9)	0.53	28.6 (1.9)	<0.01	25.7 (0.7)	0.64	26.6 (0.6)	0.65	25.2 (0.4)	0.43	22.9 (0.9)	0.26

Abbreviations: EtOH, alcohol; IVDU, intravenous drug use; PAK, pancreas after kidney transplant; PTA, pancreas transplant alone; SPK, simultaneous pancreas kidney transplant; THC, tetrahydrocannabinol.

**TABLE 2:** Donor characteristics stratified by donor substance use history and donor toxicology screen.

	*N* (%)	Ethanol	*p*	Tobacco	*p*	Marijuana	*p*	IVDU	*p*	Cocaine (any)	*p*	Cocaine (last 6 months)	*p*
**Total Cohort** (*n* (%))	737 (100%)	63 (9%)		285 (40%)		216 (31%)		18 (3%)		75 (10%)		36 (5%)	
**Gender** (*n* (%))			0.09		0.99		<0.01		0.54		0.19		0.39
Male	456 (62%)	46 (73%)		180 (63%)		153 (71%)		10 (56%)		52 (69%)		25 (69%)	
Female	267 (36%)	17 (27%)		105 (37%)		63 (29%)		8 (44%)		23 (31%)		11 (31%)	
**Race** (*n* (%))			0.36		<0.01		0.16		0.87		0.36		0.59
White	564 (78%)	53 (84%)		241 (85%)		172 (80%)		15 (83%)		64 (85%)		30 (83%)	
Black	101 (14%)	5 (8%)		30 (11%)		34 (16%)		2 (11%)		7 (9%)		3 (8%)	
Other	51 (7%)	5 (8%)		14 (5%)		10 (5%)		1 (6%)		4 (5%)		3 (8%)	
**Age** (median (SE))	24 (0.4)	33 (1.2)	<0.001	27 (0.6)	<0.001	25.0 (0.6)	0.09	30 (1.1)	0.11	28 (0.8)	<0.01	28 (1.2)	0.21
**BMI** (median (SE))	23.7 (0.2)	23.6 (0.5)	0.68	23.9 (0.2)	0.29	23.6 (0.3)	0.81	24.0 (0.9)	0.98	23.9 (0.4)	0.62	23.9 (0.6)	0.93
**Donor Type** (*n* (%))			0.95		0.65		0.83		0.57		0.01		0.05
DBD	722 (98%)	62 (98%)		282 (99%)		212 (98%)		18 (100%)		71 (95%)		34 (94%)	
DCD	15 (2%)	1 (2%)		3 (1%)		4 (2%)		0 (0%)		4 (5%)		2 (6%)	
**Cause of death** (*n* (%))			0.25		0.04		0.29		<0.01		0.03		0.07
Stroke	110 (15%)	14 (22%)		51 (18%)		26 (12%)		2 (12%)		12 (16%)		6 (17%)	
Trauma	431 (59%)	33 (52%)		171 (60%)		134 (62%)		5 (29%)		34 (45%)		15 (42%)	
Anoxic Brain Injury	191 (26%)	16 (25%)		63 (22%)		56 (26%)		11 (59%)		29 (39%)		15 (42%)	
**Transplant data**													
**Total Ischemic Hours** (median (SE))	8.3 (0.1)	8.0 (0.6)	0.12	8.0 (0.2)	0.02	8.0 (0.2)	0.3	8.1 (0.5)	0.63	7.8 (0.4)	0.98	7.8 (0.8)	0.26

Positive Donor Toxicology Screen																			
	*N* (%)	Toxico	*p*	EtOH	*p*	THC	*p*	Amp	*p*	Barbitu	*p*	Coca	*p*	Op	*p*	Benzod	*p*	Metha	*p*
**Total Cohort**	737 (100%)	463 (63%)		96 (22%)		126 (26%)		29 (6%)		10 (2%)		27 (6%)		53 (11%)		106 (22%)		5 (1%)	
**Gender**			0.04		0.93		<0.001		0.44		0.79		0.12		0.94		0.95		0.26
Male	456 (62%)	306 (66%)		64 (67%)		97 (77%)		17 (57%)		6 (60%)		21 (78%)		34 (64%)		68 (64%)		1 (20%)	
Female	267 (36%)	157 (34%)		32 (33%)		29 (23%)		12 (43%)		4 (40%)		6 (22%)		20 (37%)		38 (36%)		4 (80%)	
**Race**			0.32		0.16		<0.01		0.6		0.24		0.62		0.67		0.53		0.04
White	564 (78%)	370 (80%)		76 (79%)		89 (71%)		25 (86%)		7 (70%)		20 (74%)		44 (83%)		88 (83%)		4 (80%)	
Black	101 (14%)	65 (14%)		11 (11%)		28 (22%)		2 (7%)		1 (10%)		4 (15%)		7 (14%)		11 (10%)		0 (0%)	
Other	51 (7%)	28 (6%)		10 (10%)		9 (7%)		2 (7%)		2 (20%)		3 (11%)		2 (4%)		7 (7%)		1 (20%)	
**Age** (median (SE))	24 (0.4)	25 (0.5)	<0.01	27 (1.0)	<0.001	24 (0.8)	0.93	26 (1.9)	0.31	25 (3.5)	0.56	26 (1.4)	0.95	25 (1.3)	0.57	25 (0.8)	0.89	34 (2.8)	0.21
**BMI** (median (SE))	23.7 (0.2)	23.7 (0.2)	0.29	24 (0.4)	0.94	23.3 (0.3)	0.15	22.3 (0.8)	0.09	23.5 (1.3)	0.92	24.1 (0.8)	0.56	23.5 (0.6)	0.60	23.8 (0.4)	0.82	25.5 (1.5)	0.39
**Donor Type**			0.29		0.52		0.13		0.08		0.63		0.6		0.85		0.23		0.01
DBD	722 (98%)	454 (98%)		93 (97%)		121 (96%)		27 (93%)		10 (100%)		26 (96%)		52 (98%)		102 (96%)		4 (80%)	
DCD	15 (2%)	9 (2%)		3 (3%)		5 (4%)		2 (7%)		0 (0%)		1 (4%)		1 (2%)		4 (4%)		1 (20%)	
**Cause of death**			<0.01		<0.001		0.99		0.11		0.10		0.5		<0.001		0.82		0.02
Stroke	110 (15%)	55 (12%)		6 (6%)		15 (12%)		4 (14%)		3 (30%)		4 (15%)		2 (4%)		13 (12%)		1 (20%)	
Trauma	431 (59%)	281 (61%)		80 (83%)		74 (59%)		12 (41%)		3 (30%)		13 (48%)		23 (43%)		60 (57%)		0 (0%)	
Anoxic Brain Injury	191 (26%)	127 (27%)		10 (10%)		37 (29%)		13 (45%)		4 (40%)		10 (37%)		28 (53%)		33 (31%)		4 (80%)	
**Transplant data**																			
**Total Ischemic Hours** (median (SE))																			
	8.3 (0.1)	7.9 (0.1)	0.78	8.3 (0.5)	<0.001	7.9 (0.3)	0.94	6.7 (0.3)	0.01	8.8 (0.6)	0.79	8.5 (1.1)	0.04	7.9 (0.3)	0.77	8.0 (0.3)	0.31	8.1 (0.1)	0.91

Abbreviations: DBD, deceased after brain death; DCD, deceased after cardiac death; EtOH, alcohol; THC, tetrahydrocannabinol; IVDU, intravenous drug use.

Toxicology screens were positive for cocaine in 6% of all donors with a toxicology screen available. Toxicology screens were more often available for donors to recipients of SPK transplants (65% vs 61%, *p* < 0.001) and male recipients (60% vs 56%, *p* < 0.01). Donors from whom a toxicology screen was obtained were more frequently male (66% vs 63%, *p* = 0.04), older (25 vs 24, *p* < 0.01), and died due to trauma (83% vs 59%, *p* < 0.001).Donors with toxicology screen positive for ethanol were older (27 vs 25, *p* < 0.001), more frequently died by trauma (83% vs 61%, *p* < 0.001), and had longer total ischemia time (8.3 vs 7.9, *p* < 0.001). Allografts with marijuana positive toxicology screens were more frequently male (77% vs 66%, *p* < 0.001) and Black (22% vs 14%, *p* < 0.01). Donor toxicology screen was positive for methadone more frequently in DCD donors (20% vs 2%) and less frequently in donors who died by trauma (0% vs 61%, *p* = 0.02). Donors with toxicology screens positive for opiates more frequently died by anoxic brain injury (53% vs 28%, *p* < 0.001). Total ischemia time was significantly shorter in donors with a toxicology screen positive for amphetamine (6.7 vs 7.9, *p* = 0.01) and significantly longer in donors with a toxicology screen positive for cocaine (8.5 vs 7.9, *p* = 0.04). Recipients of allografts from donors with positive toxicology screens were similar in age and BMI, except donors with positive toxicology screens for barbiturates, which had a higher median BMI (28.6 vs 25.4, *p* < 0.01). Recipients undergoing a retransplant more often received a graft from a donor with toxicology screen positive for benzodiazepines (11% vs7%, *p* = 0.02), male recipients more often received a graft from donors with toxicology screen positive for opiates (74% vs 60%, *p* = 0.02), and Black recipients more often received a graft from donors with toxicology screen positive for marijuana (17% vs 9%, *p* = 0.02) when comparing all of those with toxicology screen collected.

### 3.2 Peak Amylase and Lipase

Post‐pancreas transplant 30‐day peak serum amylase and lipase varied by donor exposure to intoxicants. Donor history of tobacco use exceeding 20 pack‐years was associated with significantly higher peak amylase levels (median 175 U/L, *p* < 0.01), but not with peak lipase levels (*p* = 0.98). A history of marijuana use was associated with higher lipase levels (*p* = 0.05), but not significantly associated with peak amylase levels (*p* = 0.25). No other donor substance use history, including ethanol, IVDU, or any cocaine use, was significantly associated with elevated amylase or lipase levels. Positive toxicology screen for THC was associated with higher peak serum lipase levels (median 168 U/L, *p* = 0.04), but not amylase (*p* = 0.44). Other substances detected on toxicology screen, including cocaine, ethanol, amphetamines, barbiturates, opiates, benzodiazepines, and methadone, were not significantly associated with elevations in either peak lipase or amylase.

### 3.3 Hospital Length of Stay and Readmissions

Hospital length of stay and readmissions within one year after transplant by intoxicant exposure is presented in Table 3. The mean hospital length of stay (LOS) following the index transplant was 11 days (SD 16). Donor history of alcohol use was associated with longer LOS than those without (15 vs 11 days, *p* = 0.05), while no other donor history of substance use significantly impacted the length of stay. Similarly, LOS was significantly longer for those with transplants from donors with toxicology screens positive for ethanol (14 vs 10 days, *p* < 0.01). No other toxicology screen positive results were associated with significantly different LOS.

**TABLE 3:** Post‐transplant length of stay and readmissions within 1‐ year post‐transplant, stratified by donor substance use history and donor toxicology screen.

	Overall (*n* (%))	Hospital length of stay post‐transplant (mean, (SD))	*p*‐value	Any readmission (*n* (%))	*p*‐value	*N* of readmission (mean, (SD))	*p*‐value
Overall	737 (100%)	11 (16)		241 (33%)		1.0 (2.2)	
**Donor substance use history**							
Alcohol use							
Yes	63 (9%)	15 (18)	0.05	23 (37%)	0.58	1.0 (2.3)	0.94
No	674 (91%)	11 (15)		222 (33%)		1.0 (2.2)	
Tobacco use > 20 years							
Yes	285 (40%)	12 (17)	0.09	51 (18%)	<0.001	0.6 (2.1)	<0.001
No	452 (60%)	10 (14)		194 (43%)		1.3 (2.3)	
Marijuana							
Yes	216 (31%)	11 (13)	0.64	67 (31%)	0.30	1.0 (2.6)	0.72
No	521 (69%)	11 (16)		182 (35%)		1.0 (2.1)	
IVDU							
Yes	18 (3%)	13 (16)	0.62	9 (50%)	0.14	2.0 (4.0)	0.06
No	719 (97%)	11 (15)		237 (33%)		1.0 (2.2)	
Cocaine (any history)							
Yes	75 (11%)	11 (10)	0.95	30 (40%)	0.25	1.2 (3.0)	0.38
No	662 (89%)	11 (16)		218 (33%)		1.0 (2.2)	
Cocaine (within 6 months)							
Yes	36 (5%)	12 (12)	0.63	17 (47%)	0.10	1.2 (1.6)	0.64
No	701 (95%)	11 (16)		231 (33%)		1.0 (2.3)	
**Donor toxicology screen**							
Tox screen available	463 (63%)	12 (17)	0.29	176 (38%)	<0.001	1.2 (2.4)	<0.01
No tox screen	274 (37%)	10 (13)		63 (23%)		0.7 (1.8)	
EtOH							
Yes	96 (22%)	14 (21)	<0.01	33 (34%)	0.02	1.0 (1.7)	0.16
No	367 (78%)	10 (10)		176 (48%)		1.3 (2.2)	
THC							
Yes	126 (26%)	10 (12)	0.23	55 (44%)	0.84	1.2 (2.1)	0.85
No	337 (74%)	12 (18)		145 (43%)		1.3 (2.5)	
Amphetamine							
Yes	29 (6%)	7 (3)	0.15	15 (52%)	0.44	1.3 (2.1)	0.83
No	434 (94%)	12 (17)		187 (43%)		1.2 (2.5)	
Barbiturate							
Yes	10 (2%)	15 (21)	0.56	2 (20%)	0.20	2.7 (5.8)	0.06
No	453 (98%)	11 (16)		199 (44%)		1.2 (2.3)	
Cocaine							
Yes	27 (6%)	12 (10)	0.95	12 (44%)	1.00	1.2 (1.6)	0.82
No	436 (94%)	11 (17)		192 (44%)		1.3 (2.5)	
Opiate							
Yes	53 (11%)	8 (4)	0.08	16 (30%)	0.04	0.8 (2.1)	0.16
No	410 (89%)	12 (17)		185 (45%)		1.3 (2.5)	
Benzodiazepine							
Yes	106 (22%)	14 (26)	0.10	47 (44%)	0.82	1.4 (3.1)	0.42
No	357 (78%)	11 (12)		154 (43%)		1.2 (2.2)	
Methadone							
Yes	5 (1%)	7 (1)	0.51	1 (20%)	0.37	1.0 (2.2)	0.77
No	458 (99%)	10 (12)		229 (50%)		1.3 (2.0)	

Abbreviation: EtOH, alcohol; IVDU, intravenous drug use; THC, tetrahydrocannabinol.

Overall, 241 patients (33%) had at least one readmission within one year post‐transplant, with a mean of 1.0 readmissions (SD 2.2). Readmissions were lower among those who received transplants from donors with a history of tobacco use, including both any readmissions (18% vs 43%, *p* < 0.001) and readmissions within the first year (0.6 vs 1.3, *p* < 0.001). Significantly fewer of those who received transplants from donors with toxicology screen positive for opiates (30% vs 45%, *p* = 0.04) or ethanol (34% vs 48%, *p* = 0.02) were readmitted within one‐year post‐transplant, though overall there was no statistically significant difference in mean number of readmissions between those with donors with opiate‐ or ethanol‐positive toxicology screens compared to those with toxicology screens negative for those substances.

### 3.4 Pancreas Allograft Survival

Pancreas allograft survival by intoxicant exposure is presented in Table 4. Overall, 7‐day allograft loss was significantly higher in transplants from donors with history of alcohol use (8% vs 3%, *p* < 0.01). Allografts from donors with a toxicology screen positive for alcohol had significantly higher 90‐day pancreas allograft loss (8% vs 5%, *p* = 0.02) and significantly lower 1‐year allograft survival (89% vs 93%, *p* < 0.01) (Table 4). Although 90‐day pancreas allograft loss was higher in allografts from donors with a history of alcohol use (10% vs 5%, *p* = 0.08) or more than 20 pack‐years of tobacco use (7% vs 5%, *p* = 0.07), these did not reach statistical significance. There were no significant differences in allograft survival for donor history of use of cocaine, marijuana, or intravenous illicit drugs. Allografts from donors with a toxicology screen positive for benzodiazepines had worse 1‐year graft survival (91% vs 93%, *p* = 0.06), though this did not reach statistical significance. There was no significant difference in allograft survival from donors with toxicology screens positive for cocaine, marijuana, amphetamine, barbiturate, opiates, or methadone. A Kaplan Meier analysis comparing pancreas allograft survival from donors specifically with a toxicology screen positive for cocaine to those with a toxicology screen negative for cocaine (Figure 1) demonstrated that there was no significant difference in allograft survival (*p* = 0.51).

**TABLE 4:** Post‐pancreas transplant allograft survival, including 7‐ and 90‐ day allograft loss and 1‐year graft survival, stratified by donor substance use history and donor toxicology screen.

	7‐day graft loss	*p*	90‐day graft loss	*p*	1‐year graft survival	*p*	1‐year patient survival	*p*
**Overall cohort** (*n* (%))	22 (3%)		37 (5%)		700 (93%)		715 (97%)	
**Donor substance use history**								
Alcohol use	8%	<0.01	10%	0.08	90%	0.35	100%	0.17
Tobacco use > 20 pack years	4%	0.10	7%	0.07	92%	0.23	97%	0.82
Marijuana	2%	0.80	4%	0.60	94%	0.45	98%	0.37
IVDU	6%	0.45	6%	0.91	94%	0.85	100%	0.49
Cocaine use (any)	1%	0.48	4%	0.73	92%	0.62	96%	0.46
Cocaine use (within 6 months)	0%	0.32	6%	0.85	92%	0.67	97%	0.94
**Donor Toxicology Screen**								
Positive for any substance	2%	0.23	4%	0.20	94%	0.14	98%	0.65
EtOH	3%	0.39	8%	0.02	89%	< 0.01	97%	0.27
THC	2%	0.56	5%	0.67	93%	0.44	96%	0.13
Amphetamine	0%	0.40	0%	0.25	100%	0.17	100%	0.40
Barbiturate	10%	0.10	10%	0.34	90%	0.56	100%	0.63
Cocaine	0%	0.42	0%	0.27	96%	0.64	96%	0.60
Opiate	2%	0.85	2%	0.39	94%	0.98	96%	0.43
Benzodiazepine	2%	0.78	6%	0.36	91%	0.06	96%	0.23
Methadone	0%	0.75	0%	0.68	100%	0.61	100%	0.77

Abbreviation: EtOH, alcohol; IVDU, intravenous drug use; THC, tetrahydrocannabinol.

**FIGURE 1:**
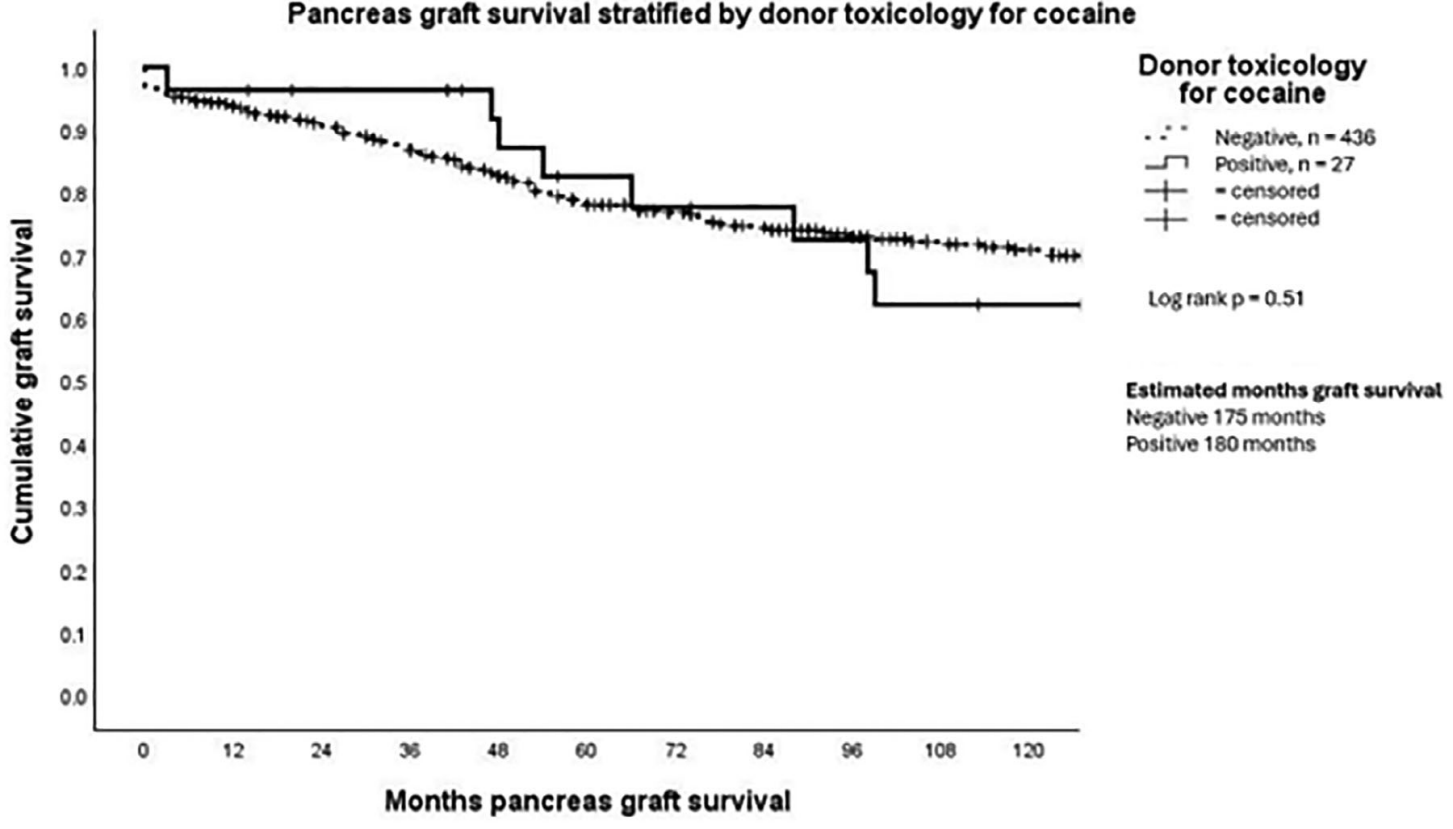
Kaplan Meier analysis comparing pancreas allograft survival from donors with cocaine positive toxicology screen to cocaine negative toxicology screen.

### 3.5 Return to the Operating Room within 1‐Year of Transplant

For the entire cohort, the rate of re‐laparotomy for any reason within 1‐year of the index transplant was 26.17%. The most common reasons for return to the operating room included small bowel obstruction for enterolysis for 61 recipients (32.1%), elective repair of an incisional hernia for 51 recipients (26.8%), and concern for graft thrombosis for 22 recipients (11.6%). Allograft pancreatitis was not a common indication for re‐exploration. There was no significant difference in the rate of re‐laparotomy for any reason within 1‐year between those with a donor history positive for any substance use versus those with a donor history negative for any substance use (26.49% vs 25.98%, *p* = 0.87). There was also no significant difference in re‐laparotomy when comparing by donor history of specific substance use, including EtOH (*p* = 0.21), tobacco (*p* = 0.60), marijuana (*p* = 0.66), IVDU (*p* = 0.60), or cocaine (*p* = 0.63).

## 4 Discussion

A substantial portion of donors of pancreatic allografts had a history of substance use or positive toxicology screens, most commonly for tobacco or marijuana, though 11% of donors also had past cocaine use, nearly half of whom had used cocaine recently. The rates of substance use amongst donors in our cohort are higher than that of the general population, in which approximately 1% of adults are estimated to use cocaine [20]. Donor and recipient characteristics varied by both reported substance use and positive toxicology findings. Black and male recipients more frequently received allografts from donors with reported substance use history or positive toxicology screen. Donor history of more than 20 pack‐years of tobacco use was associated with significantly higher peak amylase levels and donors with toxicology screens positive for THC were associated with higher peak serum lipase levels in the early postoperative period, though this was not associated with differences in allograft survival. Donor history of alcohol use was associated with significantly higher 7‐day allograft loss, while allografts from donors with positive toxicology screen for alcohol were associated with significantly higher 90‐day graft loss and significantly lower 1‐year allograft survival. Hospital length of stay was longer for those with donors with a history of alcohol use or a donor toxicology screen positive for ethanol. There were fewer readmissions in the first‐year post‐transplant for those with a donor history of tobacco use or a donor toxicology screen positive for opiates or ethanol. Donor exposure to cocaine as documented in the history or toxicology reports was not associated with any inferior outcomes. Donor substance use or positive toxicology screen was common among our cohort, consistent with rates reported previously [5, 6, 21]. As cocaine was of particular interest, we stratified reported cocaine use as any use and use within the previous 6 months, neither of which was found to significantly impact outcomes.

Though the demand for pancreas transplant continues to outweigh the available supply, there likely still exists stigma against transplanting pancreas allografts from donors with a history of substance use. Importantly, the information available regarding donor substance use at the time of organ offer is inherently limited. Toxicology screening captures only relatively recent exposure and provides no information regarding the duration, frequency, or intensity of substance use. It is likely that some donors with toxicology screen positive for benzodiazepines or opiates received these medications in the course of their pre‐hospital emergency medical care or early in their hospitalization, which would ultimately be impossible to differentiate from recreational use as documentation of the receipt or timing of administration of these medications is not available. Likewise, substance use histories obtained from next of kin may be incomplete or subject to recall bias. Consequently, transplant teams frequently make organ acceptance decisions without a clear understanding of the chronicity or severity of a donor's substance use. This study evaluates the information that is actually available to transplant clinicians during organ offer assessment.

It is also important to recognize that utilization of a pancreas allograft is not based solely on donor history or toxicology findings. Organ acceptance incorporates a comprehensive assessment of donor characteristics and direct evaluation of the allograft by procurement and transplant surgeons. Pancreas allografts that demonstrated evidence of pancreatitis, edema, fibrosis, or fatty infiltration are declined prior to transplantation and therefore are not represented in this analysis. Accordingly, our results reflect outcomes among allografts that were ultimately deemed suitable for transplantation. Within that context, a history of substance use or positive toxicology finding, in the setting of an otherwise acceptable pancreas allograft, appears to be compatible with favorable transplant outcomes.

Registry‐based studies of outcomes of pancreas transplants performed in the UK have similarly found that donor substance misuse is not associated with decreased allograft or patient survival [19, 22], however, contrary to our findings, these studies also specifically found that donor alcohol use was not associated with impaired graft survival. In our cohort, donors with reported alcohol use or positive toxicology screens for alcohol tended to be older, and donors with positive alcohol screens experienced longer ischemia times, factors that may have contributed to the observed associations with graft loss and reduced one‐year graft survival. There may also be population differences between the UK and the United States that may account for some of these discrepancies. Although fewer of those with donor toxicology positive for ethanol were readmitted in the first year post‐transplant, the mean number of readmissions did not differ significantly, suggesting that those who were readmitted had higher than average readmissions within the first year. While these findings suggest that donor alcohol exposure may warrant additional scrutiny during organ assessment, they should be interpreted within the broader context of donor and transplant characteristics, and donor use of other substances should not be considered a contraindication to transplant if the allograft otherwise seems appropriate for transplant.

There are several relevant limitations to this single institution, retrospective study. This analysis does not include allografts that were excluded from transplantation and therefore cannot assess organ non‐utilization related to donor substance use. Additionally, the downtime of donors was not included in the analysis, nor was the cause of donor death specified if secondary to chemical intoxication. Not all donors had toxicology screens available in the medical record, and thus, there may be an underreporting of positive toxicology screen results for donors. Not all donors had complete information obtained from next of kin regarding donor history of substance use.  Importantly, the number of donors with documented cocaine exposure was relatively small, limiting the statistical power to detect clinically meaningful differences in outcomes and raising the possibility of a Type II error. The absence of a statistically significant association between donor cocaine exposure and adverse outcomes should not be interpreted as evidence that no such association exists. Rather, these findings provide reassuring preliminary data regarding the use of otherwise acceptable pancreas allografts from donors with a history of cocaine exposure.

There are limited data specifically evaluating the impact of donor cocaine exposure on pancreas transplantation outcomes. In this context, despite the limitations of sample size, our cohort adds clinical experience from a high‐volume transplant center to a relatively limited body of evidence. Larger multicenter studies will be needed to better define the relationship between donor cocaine exposure and pancreas transplant outcomes and to determine whether clinically meaningful differences can be reliably excluded. Nevertheless, within the population of pancreas allografts that are otherwise deemed suitable for transplantation, our findings suggest that donor substance use history or positive toxicology findings alone should not necessarily preclude consideration of transplantation and can be compatible with favorable transplant outcomes.

## 5 Conclusion

Only a donor history of alcohol use was associated with worse outcomes following pancreas transplantation. No significant association between donor cocaine exposure and inferior outcomes was identified in this cohort. Donor substance use history or positive toxicology findings, in the setting of an otherwise acceptable pancreas allograft, should not necessarily preclude transplantation, although donor alcohol use may warrant additional consideration. Larger multicenter studies are needed to further characterize the potential impact of donor cocaine exposure on pancreas transplant outcomes.

## Funding

The authors have nothing to report.

## Disclosures

The authors have nothing to report.

## Conflicts of Interest

None of the authors have a conflicts of interest to disclose.

## Data Availability

The data that support the findings of this study are available on request from the corresponding author. The data are not publicly available due to privacy or ethical restrictions.
